# Diagnostically important muscle pathology in DNAJB6 mutated LGMD1D

**DOI:** 10.1186/s40478-016-0276-9

**Published:** 2016-02-05

**Authors:** Satu Sandell, Sanna Huovinen, Johanna Palmio, Olayinka Raheem, Mikaela Lindfors, Fang Zhao, Hannu Haapasalo, Bjarne Udd

**Affiliations:** Department of Neurology, Seinäjoki Central Hospital, Seinäjoki, Finland; Department of Neurology, Tampere University Hospital, Tampere, Finland; Neuromuscular Research Center, Tampere University Hospital, University of Tampere, Tampere, Finland; Department of Pathology, Fimlab Laboratories, Tampere University Hospital, University of Tampere, Tampere, Finland; Department of Pathology and Genetics, HUSLAB Laboratories, Helsinki University Hospital, University of Helsinki, Helsinki, Finland; Department of Medical Genetics, Folkhälsan Institute of Genetics, University of Helsinki, Helsinki, Finland; Department of Neurology, Vaasa Central Hospital, Vaasa, Finland

**Keywords:** LGMD1D, DNAJB6, Myopathy, Autophagy, CASA

## Abstract

**Introduction:**

Limb girdle muscular dystrophies are a large group of both dominantly and recessively inherited muscle diseases. LGMD1D is caused by mutated DNAJB6 and the molecular pathogenesis is mediated by defective chaperonal function leading to impaired handling of misfolded proteins which normally would be degraded. Here we aim to clarify muscle pathology of LGMD1D in order to facilitate diagnostic accuracy.

After following six Finnish LGMD1D families, we analysed 21 muscle biopsies obtained from 15 patients at different time points after the onset of symptoms. All biopsies were obtained from the lower limb muscles and processed for routine histochemistry, extensive immunohistochemistry and electron microscopy.

**Results:**

Histopathological findings were myopathic or dystrophic combined with rimmed vacuolar pathology, and small myofibrillar aggregates. These myofibrillar inclusions contained abnormal accumulation of a number of proteins such as myotilin, αB-crystallin and desmin on immunohistochemistry, and showed extensive myofibrillar disorganization with excess of Z-disk material on ultrastructure. Later in the disease process the rimmed vacuolar pathology dominated with rare cases of pronounced larger pleomorphic myofibrillar aggregates. The rimmed vacuoles were reactive for several markers of defect autophagy such as ubiquitin, TDP-43, p62 and SMI-31.

**Conclusions:**

Since DNAJB6 is known to interact with members of the chaperone assisted selective autophagy complex (CASA), including BAG3 – a known myofibrillar myopathy causing gene, the molecular muscle pathology is apparently mediated through impaired functions of CASA and possibly other complexes needed for the maintenance of the Z-disk and sarcomeric structures. The corresponding findings on histopathology offer clues for the diagnosis.

## Introduction

Limb girdle muscular dystrophy (LGMD) 1D is an autosomal dominant myopathy usually with late onset of weakness at the age of 30–60 and slow progression, although more aggressive forms can occur [1]. The disease affects mainly the lower limb muscles with a particular pattern of preferential involvement [2]. Typically the first symptom is difficulty in climbing stairs. Loss of ambulation does not occur until late senescence, except for the aggressive form of the disease [3]. Some patients may suffer from dysphagia or dysarthria. Phenotype descriptions and families from different ethnic populations have been reported [4, 5].

Myofibrillar myopathies (MFMs) are a group of myopathies that share common morphological findings but have different molecular etiologies [6, 7]. MFMs are characterized by a distinct pathological pattern of dark and hyaline cytoplasmic changes on trichrome staining corresponding to myofibrillar dissolution and accumulation of myofibrillar aggregates causing accumulated expression of several proteins, including desmin, myotilin, alphaB-crystallin and occasional amyloid material. On electron microscopy pathological alterations are marked in Z-disc structures leading to Z-line streaming and dispersion of dark Z-disc components [8]. A novel term: Protein aggregate and vacuolar myopathy, comprises a larger spectrum of myopathies unified by their pathological features, including the myofibrillar myopathies and various rimmed vacuolar myopathies with protein aggregates or inclusions [9].

We have previously reported preliminary data on muscle biopsy findings in patients with p.F93L mutation in *DNAJB6* [1, 10] and Sato et al. have reported biopsy findings in five LGMD1D patients [11] with the p.F93I mutation. In our current study of a large biopsy material of LGMD1D patients we show consistent pathological changes in the routine histopathology that can be used to direct the molecular genetic confirmation of the final diagnosis. Moreover, we show that the abnormal protein accumulations follow a sequential evolution from the early stage of small central myofibrillar lesions to the later stage of widespread myofibrillar disintegration with and autophagic rimmed vacuolar pathology in atrophic degenerating fibers.

## Materials and methods

**Fig. 1:**
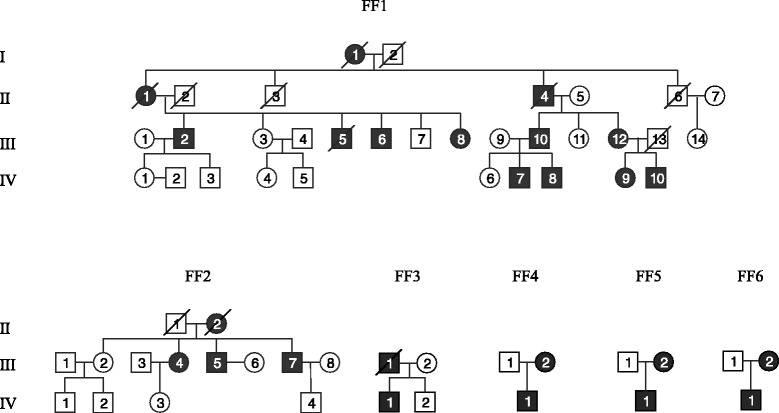
Pedigrees of Finnish families FF1–6. Black figures represent affected individuals

15 patients from six unrelated Finnish families were studied, four of them had two and one patient three biopsies, with an age range from 47 to 78 years. Patient data and their clinical findings are presented in Table 1. Clinical examination data was available from 13 patients. All patients were heterozygous for the *DNAJB6* mutation p. F93L. Pedigrees of the families FF1–6 are presented in Fig. 1.

**Table 1:** Clinical summary

Patient	Sex	Age of onset	Age at muscle biopsy	Symptoms (when examined)	CK	EMG	Other
FF1							
III-6	M	50	74	ll severe, ul mild	400	na	
III-8	F	60	69	ll mild, ul mild	187	myopathic	
III-12	F	35	47, 63, 72	ll severe, ul moderate	338	myopathic	
IV-7	M	34	46, 48	ll moderate, ul moderate	789	myopathic	dysphagia
V-8	M	30	38	ll mild, no ul	820	myopathic	
IV-9	F	30	38, 42	ll moderate, ul mild	375	myopathic	
IV-10	M	20	44, 49	ll moderate, ul mild	175	myopathic	
FF2							
III-4	F	50	63	ll mild, no ul	520	myopathic	
FF3							
IV-1	M	38	49, 51	ll, severe, ul moderate	600	myopathic	dysarthria, ll pain
FF4							
III-2	F	40	62	ll mild, slow course	“normal”	myopathic	
IV-1	M	28	31	ll mild, ul mild	1900	myopathic	
FF5							
IV-1	M	30	47	ll severe, ul mild	“normal”	myopathic	dysphagia
FF6							
IV-1	M	40	51	ll, severe, ul moderate	800	myopathic	

ll, lower limb; ul, upper limb. Clinical findings at the time of clinical examination, within one year of muscle biopsy sample time (or latest biopsy, if many). Creatine kinase (CK) normal upper limits 400 for men, 210 for women

All together 21 muscle biopsy samples were collected from four anatomically different muscles (vastus lateralis (VL), gluteus (G), soleus (S) and gastrocnemius medialis (GM)). Fresh-frozen 10 μm cryostat sections of muscle tissue were stained with routine histochemical techniques [12, 13]. Frozen sections of 6 μm thickness were cut and mounted on superFrost plus slides. Immunohistochemical staining was performed with the Ventana BechMark immunoautomate (Roche Tissue Diagnostics, Inc. AZ 85755) with antibodies and dilutions detailed in Table 2. The immunoreactions were visualized with a Ventana peroxidase based detection kit (UltraView Universal DAB detection system kit, Roche Tissue Diagnostics, Inc. AZ 85755). Stained sections were dehydrated using a gradient of ethanol and xylene and mounted with a solvent based mounting media.

**Table 2:** Antibodies used in immunohostochemical stainings, their dilutions and supplier

Antibody	Dilution	Supplier
Myotilin	1:50	Leica Biosystems, Novocastra
Alpha B crystallin	1:10	Leica Biosystems, Novocastra
Desmin	1:800	Biogenex
Dystrophin 2 (c-terminus)	1:100	Leica Biosystems, Novocastra
SMI-31	1:1000	Biosite
SMI-310	1:400	Abcam
TDP-43	1:175	Proteintech Europe
P62 (SQSTM1)	1:100	Santa Cruz Biotechnology
Ubiquitin	1:300	Dako
LAMP2	1:50	Southern Biotech
LC3	1:50	Novus Biologicals
LC3B	1:50	Cell Signaling technology
VCP	1:600	Thermo Scientific
FHL-1	1:200	Lifespan biosciences
DNAJB6	1:100	Abnova
HSPB8	1:200	Abcam
BAG3	1:800	Proteintech Europe
Proteasome	1:30	Atlas antibodies
Beta-amyloid	1:30	Leica Biosystems, Novocastra Life
Amyloid precursor protein	1:50	Biosciences
Wip1	1:50	Sigma
Dystrophin 1 (rod domain)	1:40	Leica Biosystems, Novocastra
Dystrophin 2 (c-terminus)	1:100	Leica Biosystems, Novocastra
Dystrophin 3 (N-terminus)	1:20	Leica Biosystems, Novocastra
Dysferlin	1:200	Leica Biosystems, Novocastra
α-Sarcoglycan	1:100	Leica Biosystems, Novocastra
β-Sarcoglycan	1:100	Leica Biosystems, Novocastra
γ-Sarcoglycan	1:15	Leica Biosystems, Novocastra
α-Dystroglycan	1:200	Millipore, Upstate
Caveolin 3	1:150	Santa Cruz Biotechnology
Merosin Laminin Alpha 2 Chain	1:500	USBiological
Utrophin	1:20	Leica Biosystems, Novocastra
Spectrin	1:50	Leica Biosystems, Novocastra

Antibodies applied for immunohistochemical stainings are detailed in Table 2.

Ultrastructural studies were performed on ten patients. Muscle specimens were processed for semithin and ultrathin sections according to standard methods [14] and examined with JEOL 1400 transmission electron microscope (JEOL, Japan). Electron micrographs were taken by Olympus-SIS Morada digital camera (Olympus Soft Imaging Solutions, Munster, Germany).

## Results

Clinical summary of the patients is presented in Table 1.

**Fig. 2:**
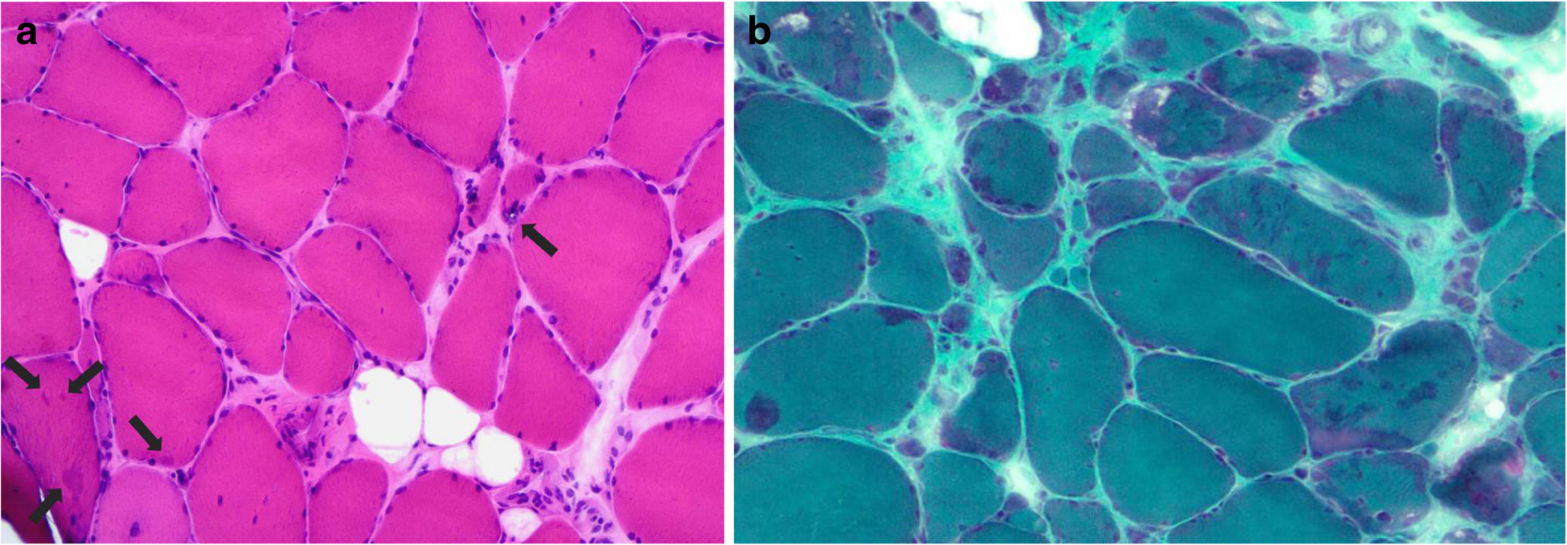
**a** On H&E staining on gastrocnemius lateralis muscle of a 51-year-old male (FF3, IV-1) mild endomysial fibrosis as well as fiber size variation, internal nuclei, one atrophic rimmed vacuolated fiber (arrow in the middle) and small centrally located myofibrillar aggregates mainly in non-atrophic fibers (arrows in left corner) are evident. **b**. In modified trichrome staining of the same muscle the aggregates appear as darker stained areas

**Fig. 3:**
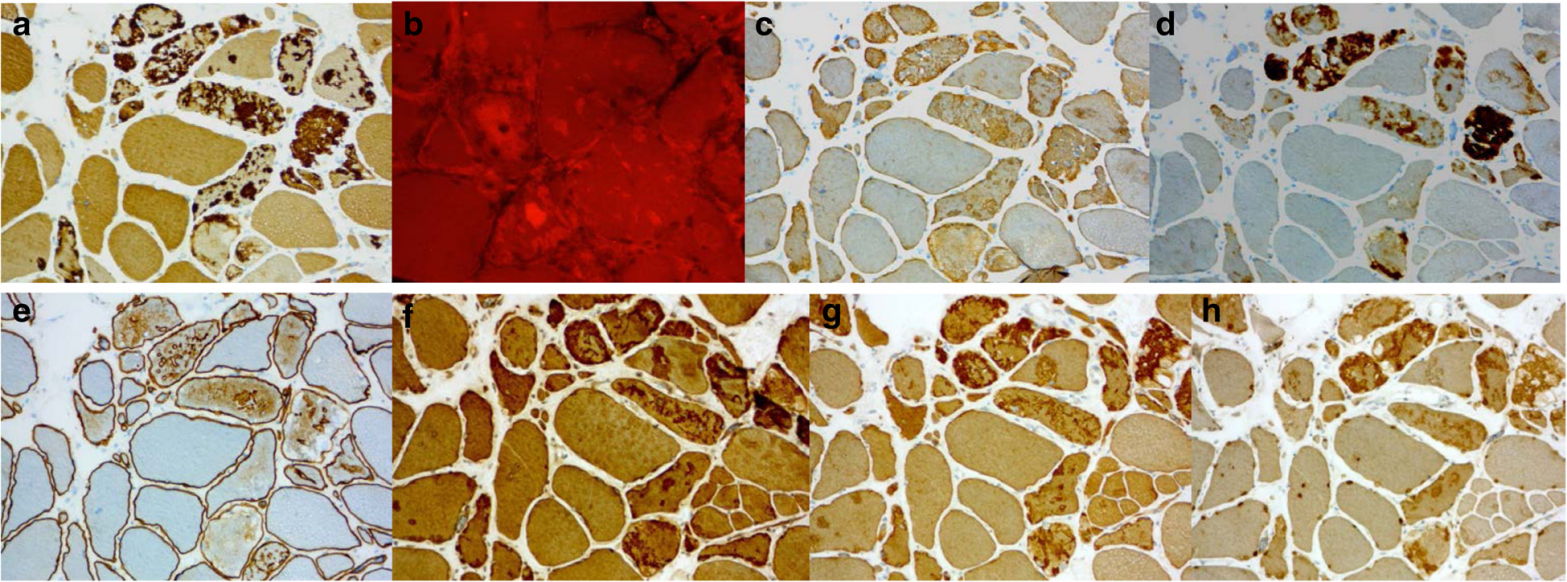
**a**, **c**, **d**. Vastus lateralis muscle of a 72-year-old female (FF1, III-12): moderate or strong expression of proteins myotilin (**a**), desmin (**c**) and alphaB-crystallin (**d**) in the myofibrillar lesions with aggregates on immunohistochemistry. **b**. Congo red staining viewed through Texas-red filters shows numerous congophilic fluorescent deposits in muscle fibers. **e**. Dystrophin-2 showed ectopic expression of mild or moderate intensity in the same regions. **f**, **g**, **h**. The protein accumulations showing CASA complex proteins BAG3 (**f**), HSPB8 (**g**), and DNAJB6 (**h**)

On light microscopy general myopathic or dystrophic features of variable severity depending on the stage of the disease were observed in all cases: fibrosis and fatty replacement of the muscle tissue, fiber size variation with atrophic and occasional hypertrophic fibers and central nucleation (Fig. 2a, b). There were occasional split fibers, necrotic and regenerating fibers, as well as focal endomysial mononuclear cells (Table 3). A uniform microscopic finding was the presence of distinct areas of myofibrillar abnormalities. In addition, rimmed vacuolar pathology was found in all cases. In the early stage pathology the myofibrillar lesions were characteristically small in size and often in a few fibers only, making them difficult to distinguish. On transverse sections they appeared as rounded dense aggregates on H&E staining and were often more easily identified on the modified trichrome stain as dark green areas. In these regions oxidative enzyme stainings were often weak, however without very distinct core or rubbed-out pathology. On longitudinal sections the myofibrillar lesions were wavy in shape extending over several sarcomeres in length. They occurred mainly in fibers with normal caliber and were usually not located in the periphery of the fibers. Both fiber types were equally involved. In the late stage of the disease the rimmed vacuolar pathology was usually very prominent. In a single biopsy a large number of fibers containing pleomorphic hyaline myofibrillar masses reminiscent of a classic myofibrillar myopathy were seen (Fig. 3a). These pleomorphic regions were occasionally congophilic on Congo red staining appearing bright red visualized through Texas red fluorescent filters (Fig. 3b). Increased expression of beta-amyloid precursor protein (APP) was also observed in some of these fiber regions associated with the hyaline deposits.

**Table 3:** Myopathological findings

		Muscle	Neurogenic fiber type grouping	Fiber type predominance	Fiber size variation	Fibrosis and fatty degeneration	Increase of internal nuclei	Fiber splitting	Necrosis	Lymphocyte infiltrates	Fiber regeneration	Rimmed vacuoles	Cytoplasmic myofibrillar inclusions	Core-like lesions	Rubbed-out fibers	Moth-eaten	Lobulated fibers	Ragged red fibers	COX– fibers
FF1																			
	III-6	VL	–	+		++	+	+	–	–	–	+	+	+	–	++	+	–	–
	III-8	S	(+)	–	++	++	++	++		–	–	+	++	++	–	–	–	–	–
	III-12	VL	–	+	(+)	+	–	–	–	–	+	(+)	+	–	–	–		–	–
		GLU	–	+	(+)	+	–	+	–	+	+	+	++	+	–	–		–	–
		VL	–		(+)	+	+	–	+	+	+	+	++	–	–	+	–	–	–
	IV-7	GM	–	–	+	+	+	+	+	+	–	+	+	+++	–	–	–	–	–
		GM	–	–	++	++	++	++	–	+	+	++	++	+	–	+	–	–	–
	IV-8	GM	–	–	+	–	+	+	+	+	+	+	+	+	–	+	+	–	–
	IV-9	VL	–	+	+	–	+	+	+	+	+	+	+	–	–	+	–	–	–
		VL	+	–	++	+	+	–	–	–	–	+	+	–	–	–	(+)	–	(+)
	IV-10	VL	–	–	–	–	–	–	–	–	–	+	(+)	+	–	–	–	–	–
		VL	–	+	+	–	+	–	–	–	–	+	+	+	–	–	–	–	–
FF2																			
	III-4	VL	–		+	–	+	–	–	–	–	+	+	–	–	–	–	–	(+)
	III-5	VL	–		+	–	+	–	–	–	–	+	++	–	–	–	–	–	(+)
	III-7	VL	–		++	++	+++	+	+	+	+	+	+	+	–	+	–	–	–
FF3																			
	IV-1	VL	+	–	++	+	+++	+	+	+	–	+	++	+	–	+	–	–	–
		GL	–	–	++	+	++	+	++	+	–	+	++	++	–	+	+	–	(+)
FF4																			
	III-2	VL	–	+	++	++	++	+	+	–	–	+	+	+	–	+	–	–	–
	IV-1	VL	–	–	(+)	–	+	–	+	–	+	+	+	++	–	+	–	–	–
FF5																			
	IV-1	VL	–	+	++	++	++	+	+	+	–	++	+	+	–	–	+++	–	–
FF6																			
	IV-1	VL	+	–	++	++	+	–	–	–	–	+	+	–	–	+	+	–	(+)

Internal nuclei amount assessed as: –, absent; (+), existent in < 1 %; +, existent up to 5 %; ++, existent in 6–20 %; +++, existent in over 20 % of the fibers. Fibrosis and fatty degeneration: –, no fibrosis, fatty degeneration; +, mildly increased; ++, moderately increased

**Fig. 4:**
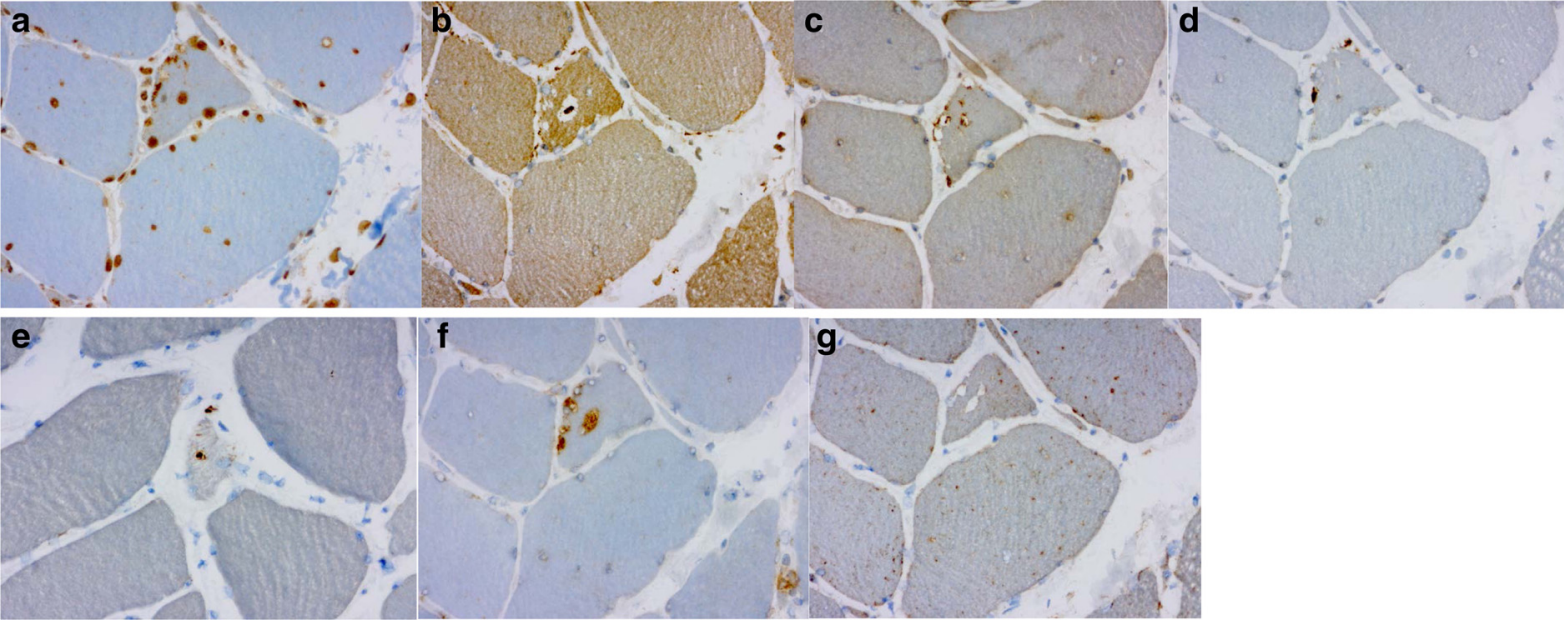
**a**, **b**, **c**, **d**, **e**. The same patient and muscle as in Fig. 3. The rimmed vacuoles showed material with reactivity for several markers of defect degradation and autophagic processing such as ubiquitin (**a**), VCP (**b**), TDP-43 (**c**), p62 (**d**) and SMI-31 (**e**). **f**, **g**. The rimmed vacuolar regions of local degeneration filled with autophagosomes, shown by LC3 (**f**) reactivity, components of mature lysosomes expressing LAMP2 (**g**)

On immunohistochemistry moderate or strong expression of several proteins including myotilin, desmin, alphaB-crystallin, proteasome and SMI310 were shown in the myofibrillar lesions with aggregates (Fig. 3a, c, d) (Table 4), and dystrophin showed ectopic expression of mild or moderate intensity in the same regions (Fig. 3e). The extent of the aggregate pathology was variable depending on the stage of the disease. The protein accumulations also contained all tested CASA complex proteins such as BAG3, heat shock protein (HSP) B8, and DNAJB6 itself (Fig. 3f, g, h). The rimmed vacuoles showed material with reactivity for several markers of defect degradation and autophagic processing such as ubiquitin, valosin-containing protein (VCP), TDP-43, p62 and SMI-31 (Table 4) (Fig. 4a, b, c, d, e). These rimmed vacuolar regions of local degeneration were filled with autophagosomes as shown by LC3 reactivity, whereas components of mature lysosomes expressing LAMP2 were less abundant or absent in the rimmed vacuolar spaces (Fig. 4f, g). Antibodies applied for the sarcolemmal proteins showed normal findings in the sarcolemma (Table 2).

**Table 4:** Immunohistochemical findings

		Muscle	Myotilin	α-BC	Desmin	DYS-2	SMI-31	TDP-43	p62	Ubiquitin	LAMP-2	VCP	FHL1	Membr prot	LC3	HSPB8	DNAJB6	Ubiquilin2	Cathepsin B	Filamin C	BAG3	α-actinin	Teletonin	Actin	SERCA	α-tubulin	SMI310	Proteasome	β-amyloid	APP	WIP1
FF1																															
	III-6	VL			+				–	–																	+		–	–	–
	III-8	S	++	++	+	++		++	++			+																			
	III-12	VL																													
		GLU			+	+																									
		VL	+	+	+	+				–		–		–																	
	IV-7	GM	++	++	+	++				++																					
		GM	++	++	++	++	++	++	++	++	–	++	–		++			++	–	–	++	+		–			++	+	–	–	–
	IV-8	GM	++	++	++	+		++	++	++	–	–	–		++			–	–	–	++	+		–		–	++	+	–	–	–
	IV-9	VL	–	+	+	+								–													–	+	–	–	–
		VL	+	+	+	–		++	++			–									++	+	+	–	–	–					
	IV-10	VL	++	++	+	+	–		+	+		–		–																	
		VL	++	++	+	+	–	+	+	++	–	–			+				–	–		–	–	+	+	–	+		–	–	–
FF2																															
	III-4	VL			–		–																								
	III-5	VL			–		–			–																					
	III-7	VL			+	+	+			+																					
FF3																															
	IV-1	VL				–				++				–																	
		GL	++	++	++	+				++				–																	
FF4																															
	III-2	VL	++	++	++	++	++	++	++	++	–	++	–		++	+	+	++	+	–	++							+	–	+	
	IV-1	VL			+	++				++																					
FF5																															
	IV-1	VL	++	++	+	+	++	++	++	–	–	–	–			+	+				+						++		–	–	–
FF6																															
	IV-1	VL	+	–	+	–	+/–	+/–	+	+	–	+	–		+		+	+/–	–												

–, normal, no immunoreactivity; +, present immunoreactivity; ++, prominent immunoreactivity

**Fig. 5:**
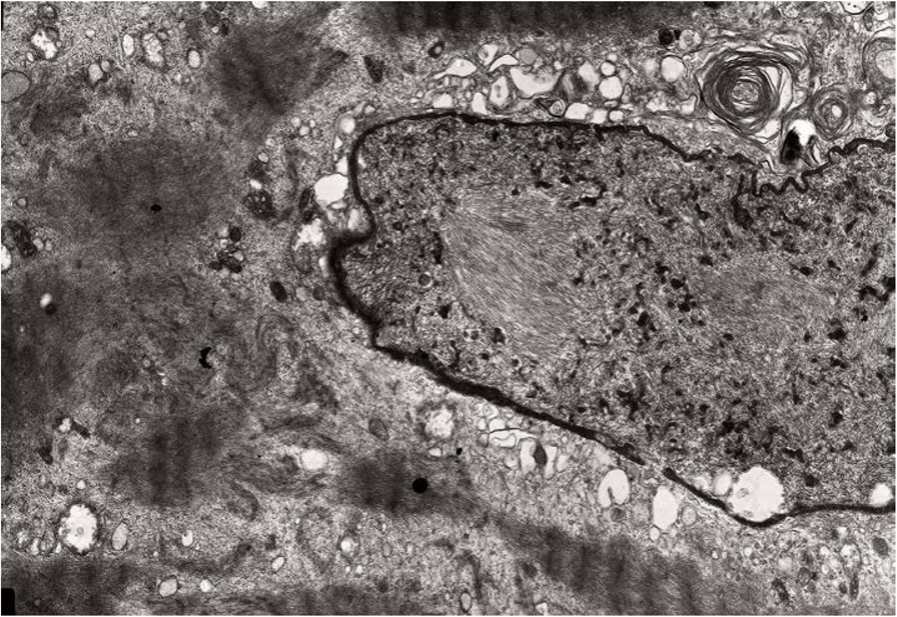
Electron micrograph from vastus lateralis muscle biopsy of a 69-year-old female (FF1, III-8) shows loss of the normal sarcomeric structure with excess dispersed wavy Z-disk material

On electron microscopy the myofibrillar lesions showed severe structural sarcomere disarrangement associated with prominent excess and dispersion of disintegrated Z-disk material (Fig. 5). Other frequent findings included autophagic regions with vesicles, myeloid figures and debris material, and fibers with multiple small vacuoles appearing to arise from the sarcoplasmic reticulum. A rare finding was the presence of occasional tubulofilamentous intranuclear or cytoplasmic inclusions in two cases.

## Discussion and conclusions

The muscle pathology in our study of *DNAJB6* mutated LGMD1D shows distinct findings that should provide clues for a correct diagnostic approach even in a sporadic patient without known family history. On light microscopy the most common finding is the presence of early stage myofibrillar disintegration with small myofibrillar aggregates and rimmed vacuolated fibers. On immunohistochemistry many proteins are found in the aggregates, and the rimmed vacuoles are reactive for several proteins associated with autophagy. The light microscopy findings are in general similar to MFMs. However, the myofibrillar aggregates are commonly distributed in scattered fibers and are smaller than in a typical MFM. Only in one biopsy large and pleomorphic aggregates in groups of fibers unevenly distributed across the fascicles more characteristic of a typical MFM were seen. In addition, rubbed-out fibers commonly found in MFMs such as desminopathies and alphaB-crystallinopathies are not a common feature in LGMD1D.

Secondary inflammatory changes that often may occur in genetic myopathies were observed in approximately half of the *DNAJB6* mutant biopsies. However, the endomysial inflammatory cells were few in number without larger infiltrates and no invasion in non-necrotic fibers was observed. In addition, there was no significant expression of MHC HLA Class I, thus clearly preventing any confusion with sporadic inclusion body myositis (s-IBM) despite the rimmed vacuolation. In addition, no significant numbers of cytochrome oxidase-negative muscle fibers or ragged red fibers were present. Clinically, the involvement of hamstrings was always more severe than the quadriceps and without the finger flexor weakness as with s-IBM.

The ultrastructural findings in myofibrillar myopathies may show some differences in the genetically different myopathies [15]. Compared to myotilinopathy the changes in LGMD1D show less frequently tubulofilamentous bundles and no basement membrane thickening, described in myotilinopathy [15]. In zaspopathy filamentous accumulations in bundles are frequent and intrasarcoplasmic rods common, but were not observed in LGMD1D. Desminopathies characteristically show cytoplasmic electrondense reticular granulofilamentous accumulations, in addition to areas with sarcomere disorganization and Z-disk alterations. Areas of amyloid-like material often adjacent to granulofilamentous inclusions or in vacuolated fibers can also be found in desminopathy, as well as other myofibrillar myopathies [8, 16]. Reticular desmin aggregates or amyloid deposits were not found ultrastructurally in the studied LGMD1D biopsies. The large complexes of electrodense granulofilamentous accumulations and sandwich formations in desminopathy and αB-crystallinopathy and filamentous bundles and extensive Z-disk alterations in myotilinopathy and zaspopathy were not observed ultrastucturally in LGMD1D.

The autophagic degenerative component of the pathology in LGMD1D with the basic features of rimmed vacuoles on light microscopy and disorganized myofibrillar structures, Z-line dispersion, and dilatation of sarcoplasmic reticulum into vacuolar formations on ultrastructure are not particularly different compared to the similar pathology of the other myofibrillar myopathies or s-IBM. However, eosinophilic inclusions in the rimmed vacuoles that occasionally may be observed in s-IBM were rare in the LGMD1D samples.

LGMD1D is caused by mutations in *DNAJB6* gene [10]. DNAJB6 belongs to the evolutionarily conserved DNAJ / HSP40 family of proteins, which regulate molecular chaperone activity by stimulating ATPase activity [17]. *DNAJB6* is expressed in many tissues [18] with its highest expression in brain. Its association to skeletal muscle disease was shown in 2012 [10]. Our previous studies suggested DNAJB6 involvement in the CASA pathway, a major mechanism in protein re-cycling and turnover for the maintenance of Z-disc sarcomeric integrity. We have in our previous work found that DNAJB6 interacts with several members of CASA complex: BAG3, HSPA8, STUB1 and HSPB8 [10]. In this study we also demonstrate in patient muscle biopsies the involvement of these proteins, thus confirming the results shown with the F93I mutation [11]. The dominant toxic effect of the mutant F93L decreases the anti-aggregational effect of DNAJB6 and makes the whole chaperonal complex in which DNAJB6 is involved less effective, leading to protein aggregations and secondary autophagic abnormalities with rimmed vacuolar pathology. Because of the defective DNAJB6 function Z-disc components are disturbed and accumulate first (myotilin, desmin, αB-crystallin, BAG3) followed by defective autophagy which can be seen as staining of rimmed vacuolar markers LC3, VCP, TDP-43, SMI-31 and p62 increasing with disease duration. These findings, when observed in the clinical diagnostic praxis, in particular the early changes of smaller myofibrillar aggregates in normal sized fibers should direct the further diagnostic efforts towards *DNAJB6* and LGMD1D disease.

Exceptionally, LGMD1B has been reported to cause myofibrillar changes similar to LGMD1D, however without rimmed vacuolar pathology [19].

Histopathology of LGMD1D is different from the other more prevalent autosomal dominant limb-girdle muscular dystrophies; markedly different from the usually nonspecific pathology in LGMD1B and LGMD1C and also distinct from the more closely related LGMD1A myotilinopathy.

## Ethics approval and consent to participate

Authors take full responsibility of the data, the analyses and the interpretation. All patients have given their informed consent prior to their inclusion in the study. All investigations have been performed in accordance of with Declaration of Helsinki and its amendments. This study has been approved by the ethical committee of Tampere University Hospital. The authors declare that they have no conflicts of interest.

## Abbreviations

BAG3: Bcl-2 associated anthanogene 3
DNAJB6: Dnaj homolog subfamily B member 6
FHL-1: Four and a half LIM domains protein 1
HSBP8: Heat shock 22 KD protein 8
LC3: Microtubule-associated protein light chain 3
LC3B: Microtubule-associated protein light chain 3, isoform B
P62/ SQSTM1: Sequestosome 1
SMI-31: Phosphorylated neurofilament
SMI-310: Neurofilament
TDP-43: TAR DNA binding protein 43
VCP: Valosin containing protein
Wip1: Wildtype p53-induced phosphatase 1
